# A Word for Woman

**Published:** 1875-11

**Authors:** 


Dr. Holland in offering a kind word for woman says:

We do not hesitate to say that the average woman, educated in the better class of schools in this country, is a better scholar and a more capable and accomplished person than the average college graduate of the other sex. What we want is cheaper schools of an equal excellence. The farmer's boy goes to college, finds cheap tuition, wins a scholarship perhaps, boards in commons, earns money during vacation, and gets through, while his sister stays at home, because the only places where she can get an equal education are expensive, beyond her means. There is no college that needs to be so richly endowned as a woman's college. Women are not men, quarrel with the fact as we may, and they cannot get along so cheaply and with such self helpfulness as men while going through the processes of their education. If we are to have women's colleges, we must have well-paid professors, philosophical apparatus, cabinets, collections, art galleries, laboratories, and they must be provided for by private munificence. Provision should be made for the poor, so that high education shall come within the reach of all. There is not a women's college, or an advanced public institution for the education of women, that is not to-day in need of a large endowment for the purpose of bringing its advantages within the reach of those whose means are small.

Now we commend the matter particularly to rich women. There are many scattered up and down the country, who are wondering what they shall do with their money when, and even before, they die. To all those we beg the privilege of commending this great object. Let the boys alone. They have been pretty well taken care of already, and the men will look after them. It is for you, as women, wishing well for your own sex, and anxious for its elevation in all possible ways to endow these institutions that are springing up about the country in its interest, so that the poor shall have an equal chance with the rich. You can greatly help to give the young women of all classes as good a chance as their brothers enjoy, and you can hardly claim a great deal of womanly feeling if you do not do it.

---

To Clean Looking-Glasses.—Take a newspaper, or part of one, according to the size of the glass. Fold it small, and dip it into a basin of clean cold water; when thoroughly wet, squeeze it out in your hand as you would a sponge, and then rub it hard all over the face of the glass, taking care that it is not so wet as to run down in streams. In fact, the paper must only be completely moistened, or damped all through. After the glass has been well rubbed with wet paper, let it rest for a few minutes, and then go over it with a fresh dry newspaper (folded small in your hand) till it looks clear and bright, which it will almost immediately, and with no further trouble. This method, simple as it is, is the best and most expeditious for cleaning mirrors, and it will be found so on trial—giving a cleanness and polish than can be produced by no other process. It is equally convenient, speedy and effective. The inside of the window-frames may be cleaned in this manner to look beautifully clear—the windows being first washed from the outside; also the glasses of spectacles, &c. The glass globe of an astral lamp may be cleaned with a newspaper in the above manner.

---

Unpunctual People.—What bores they are!—what havoc they make with the precious moments of orderly, systematic men of business! A person who is faithless to his appointments may not intend to swindle people, but he does. To those who know how to turn time to advantage, every hour has an appreciable pecuniary value; minutes, even, are worth so much apiece. He who robs you of them might just as well take so much money from your purse. The act is petty larceny or grand larceny, according to the amount of time he compels you to waste, and the value of it, at a fair appraisal, to yourself or your family. The only capital of a large portion of the community is time; their compensation is measured by the clock. The moments of which promise-breakers cheat them may represent in fact the necessaries of life; and the loss of an hour may involve the privation of a loaf, or a joint, or some other article urgently needed at home. Nobody places any confidence in persons who are habitually behind time. They scarcely succeed in any enterprise. Therefore, for your own sake, as well as for the sake of others—be punctual.

